# Enzymatic Activity and Organic Acid Profile of Phosphate-Solubilizing Bacterial Inoculants and Their Agronomic Effectiveness in Soybean

**DOI:** 10.3390/microorganisms13092016

**Published:** 2025-08-29

**Authors:** Luana Rainieri Massucato, Mayara Barbosa Silva, Mirela Mosela, Lycio Shinji Watanabe, Leandro Afonso, Antoni Wallace Marcos, Alison Fernando Nogueira, Nicholas Vieira de Sousa, Ricardo Cancio Fendrich, Marcos Ventura Faria, Leandro Simões Azeredo Gonçalves

**Affiliations:** 1 Agronomy Department, Universidade Estadual de Londrina (UEL), Londrina 86051-990, PR, Brazil; antoni.marcos03@uel.br (A.W.M.); alllisonfernando@gmail.com (A.F.N.); 2 NODUSOJA, Colombo 83407-330, PR, Brazil; mayara@nodusoja.com.br (M.B.S.);; 3 BIOINPUT, Cambé 86181-570, PR, Brazil; moselamirela@gmail.com (M.M.); shinjiwatanabe2003@gmail.com (L.S.W.); leandro.afonso@uel.br (L.A.); 4 Agronomy Department, Universidade Estadual de Maringá (UEM), Maringá 87020-900, PR, Brazil; nicholasvieira2011@gmail.com; 5 Agronomy Department, Universidade Estadual do Centro Oeste (Unicentro), Guarapuava 85015-430, PR, Brazil; ventura_faria@yahoo.com.br

**Keywords:** *Glycine max* (L.), microbial biofertilizers, plant growth promotion, nutrient cycling, phosphorus use efficiency

## Abstract

Low phosphorus (P) availability in tropical soils is one of the main constraints to agricultural productivity and the sustainability of cropping systems. In this study, we evaluated the functional potential of four bacterial strains, including those present in two commercial inoculants: Nodubiophos (Ag87-CCT 8090 and Ag94-CCT 8108), and Biomaphos (B119 and B2084), focusing on their production of phosphatase and phytase enzymes, organic acids, and their agronomic efficacy in soybean cultivation. In vitro assays showed that all strains exhibited phytase and both acid and alkaline phosphatase activities, with B2084 and Ag94 standing out in phytase-mediated mineralization. In contrast, B119 and B2084 showed the highest phosphatase activity. Organic acid production varied among strains and was influenced by the phosphate source, indicating a highly responsive metabolic regulation. Strains Ag87 and Ag94 were particularly effective in producing lactic, malic, and gluconic acids, displaying distinct profiles modulated by the available P source. In field trials, combined inoculation with Ag87 and Ag94 led to increased soybean yield, achieving performance comparable to conventional fertilization at 50% and 100% of the recommended P rate, despite applying only 30% of the total P. The results highlight complementary metabolic strategies among the evaluated strains, with the ability to solubilize and mineralize phosphorus through different mechanisms. They support their potential use as bioinoculants to enhance nutrient use efficiency and reduce fertilizer dependency in soybean cultivation.

## 1. Introduction

Soybean (*Glycine max* (L.) Merr.) is considered one of the most important crops worldwide, accounting for 71% of the global supply of plant-based protein meal and 31% of vegetable oil in the 2024/2025 season [1]. The estimated production for this season is 420 million tons, with Brazil, the United States, and Argentina responsible for 80% of global output (40, 28, and 12%, respectively) [2]. In Brazil, the soybean complex (grains, meal, and oil) accounts for 40.4% of the country’s agribusiness exports, representing approximately USD 67 billion in revenue [3].

Despite the high yield potential of soybeans in Brazil, there are growing concerns about productivity losses caused by abiotic factors [4,5,6]. Among these, nutrient stress is particularly critical, as most Brazilian soils are characterized by high acidity, elevated aluminum concentrations, and low nutrient availability, especially phosphorus (P) [7]. The limited availability of P is mainly due to the fixation of inorganic phosphorus on the reactive surfaces of iron (Fe) and aluminum (Al) oxides, which are abundant in tropical soils [8,9].

An alternative to minimize P deficiency in tropical soils is the application of soil amendments and phosphate fertilizers, essentially adapting the soil to meet the plant’s needs [10]. However, approximately 72% of the phosphate fertilizers used in Brazilian agriculture are imported, making the production system highly vulnerable to geopolitical fluctuations and exchange rate volatility [11,12]. Furthermore, this approach is inefficient, as only about 10–30% of the applied P is absorbed by plants. A large portion remains in the soil, forming a reservoir of residual P, known as “legacy P”, that accumulates in forms not readily available to plants [9,13].

Strategies to improve phosphorus use efficiency (PUE) are essential for reducing the demand and dependence of phosphate fertilizers in Brazilian agriculture [8,11,14]. Key approaches include correcting soil acidity through liming, adopting integrated crop rotation and succession systems, using cover crops during the off-season, and implementing no-till systems combined with precision agriculture techniques for optimized fertilizer application timing and dosage [11,15]. In addition, strategies such as developing cultivars with enhanced P uptake and utilization efficiency [16], as well as inoculating phosphate-solubilizing microorganisms (PSMs) [10,11,17], have gained increasing attention and are being widely studied and implemented in Brazilian agriculture [18].

PSMs play a key role in soil P cycling by converting its insoluble forms into bioavailable orthophosphate, thereby contributing to improved phosphorus use efficiency (PUE) in agricultural systems [19,20,21]. These microorganisms employ multiple mechanisms to enhance P availability, with the production of organic acids and hydrolytic enzymes (phosphatases and phytases) being the most prominent. Microbes secrete organic acids such as citric, oxalic, and malic acids into the rhizosphere, where they acidify the surrounding environment and promote phosphorus solubilization through proton substitution and metal ion complexation [22,23]. Phosphatases and phytases, in turn, mediate the release of organic phosphorus. Phytases specifically catalyze the hydrolysis of phytic acid, the primary organic P reserve in soils, releasing inorganic phosphate and less phosphorylated myo-inositols. Acid and alkaline phosphatases complement this process by hydrolyzing a broader range of organic phosphorus compounds, thereby increasing the overall bioavailability of P to plants [20,24,25,26].

Massucato et al. [10] identified two bacterial strains isolated from the maize rhizosphere, Ag87 (*Priestia megaterium*) and Ag94 (*Lysinibacillus* sp.), with high potential for plant growth promotion and phosphate solubilization in maize. Co-inoculation of these strains led to average increases in maize yield that surpassed the control treatments under reduced P fertilization (30% and 50% of the recommended dosage), with results matching the yield obtained with the full conventional P dose. Despite the promising results in maize, there is still no information available on the mechanisms involved in phosphorus solubilization and mineralization by these strains, nor on their effectiveness in promoting soybean growth, a crop of high economic importance for Brazilian agriculture. Moreover, these strains improved the plants’ P uptake efficiency, highlighting their potential to enhance this nutrient acquisition. Therefore, the present study aimed to assess the ability of these bacterial strains to produce phytase, acid and alkaline phosphatases, and organic acids, as well as to evaluate the combined effect of their inoculation on soybean growth promotion in field experiments.

## 2. Material and Methods

### 2.1. Bacterial Isolates

Four bacterial strains were evaluated in this study. Strains Ag87 (*Priestia megaterium*) and Ag94 (*Lysinibacillus* sp.) are components of the commercial product Nodubiophos, developed by the company Nodusoja (Colombo, Brazil), and are deposited in the Tropical Culture Collection of the André Tosello Foundation (https://fat.org.br), identified as CCT 8090 and CCT 8108, respectively. These strains were initially isolated from maize rhizosphere soil, and their genomic features and biotechnological potential are described in Massucato et al. [10]. The other two strains, *Priestia megaterium* CNPMS B119 (B119) and *Bacillus subtilis* CNPMS B2084 (B2084), belong to the Multifunctional Microorganisms and Phytopathogens Collection (CMMF) of Embrapa Maize and Sorghum [27] and are components of the commercial product Biomaphos^®^, developed by the company Bioma (Fazenda Rio Grande, Brazil).

### 2.2. In Vitro Analysis

#### 2.2.1. Intra- and Extracellular Phytase Production

The intra- and extracellular phytase production analysis was performed according to the protocol described by Bhandari et al. [28], with modifications. Bacterial strains were cultured in NBRIP medium (National Botanical Research Institute’s Phosphate Growth Medium) [29] and incubated on an orbital shaker at 30 °C and 150 rpm for 96 h. After incubation, cultures were centrifuged at 5000 rpm (≈419× *g*, rotor radius: 1.5 cm) at 4 °C for 30 min. The supernatant was used to assess extracellular phytase activity, while the cell pellet was processed for intracellular phytase analysis. The cell pellet was resuspended in 2 mL of 50 mmol L^−1^ acetate buffer (pH 5) containing 2 mmol/L CaCl_2_, frozen at −80 °C for 30 min, and subsequently thawed in a water bath at 42 °C for 15 min. The resulting cell suspension was sonicated for 3 min and then centrifuged at 10,000 rpm (≈1677× *g*, rotor radius: 1.5 cm) at 4 °C for 30 min. The supernatant obtained from cell lysis was collected and refrigerated for intracellular phytase determination.

To determine intra- and extracellular phytase activity, 100 µL of 0.2 mol/L acetate buffer (pH 4.5) and 80 µL of 0.25 mol/L sodium phytate solution were added to 15 mL tubes and vortexed for 10 s. Then, 200 µL of the sample was added and incubated at 37 °C for 20 min. After incubation, 800 µL of 10% (*v*/*v*) trichloroacetic acid (TCA) and 900 µL of the AAM reagent mixture (1 mol/L ascorbic acid, 0.6 mol/L H_2_SO_4_, and 0.5% ammonium molybdate in a 1:1:1 *v*/*v*/*v* ratio) were added. Samples were homogenized and incubated at 37 °C for 20 min, protected from light. All analyses were performed in triplicate for each sample, using acid washed glassware, with measurements also conducted in triplicate and results expressed as the mean of three readings. Absorbance was measured using a spectrophotometer at 820 nm, and phytase activity was expressed in mU/mL.

#### 2.2.2. Phytate Mineralization

Phytate mineralization by bacterial strains was determined the same way using the supernatant from the culture medium employed for extracellular phytase quantification but supplemented during bacterial growth with inositol hexaphosphate (phytic acid) [28]. In a Falcon tube (Thermo Fisher, Waltham, MA, USA), 200 µL of each sample was added to 800 µL of 10% TCA, followed by homogenization. Subsequently, 900 µL of the AAM solution was added, the mixture was homogenized again, and samples were incubated at 37 °C for 20 min. All assays were performed in triplicate for each sample, with triplicate measurements and results expressed as the mean of three readings. Absorbance was measured at 820 nm using a spectrophotometer, and phytate mineralization was expressed in mg/L.

#### 2.2.3. Acid and Alkaline Phosphatase Production

Acid and alkaline phosphatase activity was determined according to the method described by Tabatabai and Bremner [30]. The respective culture media were included for background measurements, avoiding potential interference in constructing calibration curves. Bacterial strains were grown in NBRIP medium supplemented with three different phosphate sources (NBRIP + Ca_3_(PO_4_)_2_, NBRIP + FePO_4_, or NBRIP + AlPO_4_), and cultures were then centrifuged at 4500 rpm (≈340× *g*, rotor radius: 1.5 cm) for 10 min. Next, 1.0 mL of the resulting supernatant or control solution was transferred to Falcon tubes. Then, 4.0 mL of the appropriate buffer solution was added: 0.1 mol/L acetate buffer (pH 5) for acid phosphatase or 0.1 mol/L Na_2_CO_3_/NaHCO_3_ buffer (pH 10) for alkaline phosphatase. The mixture was homogenized by vortexing, and 0.29 mL of 0.05 mol/L p-nitrophenyl phosphate (pNPP) substrate solution was added. Samples were incubated at 37 °C for 1 h. After incubation, 1.0 mL of 0.5 mol/L CaCl_2_ and 4 mL of 0.5 mol/L NaOH were added, followed by vortexing and centrifugation at 4500 rpm (≈340× *g*, rotor radius: 1.5 cm) for 10 min. All assays were performed in triplicate for each sample, with measurements also conducted in triplicate, and results were expressed as the mean of the three readings. Absorbance was measured using a spectrophotometer at 400 nm, and phosphatase activity was expressed in µg pNPP/mL/h.

#### 2.2.4. Organic Acid Production

The determination of organic acids was adapted from the method described by Yan et al. [31]. Bacterial strains were cultured in NBRIP medium supplemented with three different phosphate sources (NBRIP + Ca_3_(PO_4_)_2_, NBRIP + FePO_4_, or NBRIP + AlPO_4_). Aliquots of the liquid culture media were collected and analyzed for organic acid content using high-performance liquid chromatography (HPLC) on a Shimadzu LC-20A system (Kyoto, Japan). The system consisted of a high-pressure pump (LC-20AT), an autosampler (SIL-20AC HT), a refractive index detector (RID-10A), a photodiode array detector (SPD-M20A), a column oven (CTO-20A), and a system controller module (CBM-20A). Chromatographic separation was performed using a Phenomenex 5 µm C18 MG column (250 × 4.6 mm) (Phenomenex, Inc., Torrance, CA, USA). The mobile phase was a 25 mM sodium phosphate buffer adjusted to pH 2.4, and elution was carried out isocratically at a flow rate of 1.0 mL/min. The column temperature was maintained at 40 °C, and the injection volume was 20 µL. Detection was performed simultaneously using the refractive index detector (RID-10A) and the photodiode array detector (SPD-M20A), at a fixed wavelength of 205 nm and a spectral scan from 200 to 400 nm. Data acquisition and processing were carried out using Shimadzu’s LC solution software version 1.21 SP1 (Kyoto, Japan). Organic acid standards included gluconic, malic, lactic, acetic, citric, and succinic acids.

### 2.3. Field Trials

#### 2.3.1. Preparation of Bacterial Isolates

The bacterial strains, preserved in cryotubes containing tryptic soy broth (TSB) and glycerol at a 2:1 ratio and stored at −80 °C, were reactivated before inoculum preparation. Reactivation was performed by streaking the strains onto Petri dishes containing Luria-Bertani agar (LB) and incubating them at 28 °C for 24 h. A pre-inoculum for each strain was prepared by suspending isolated colonies in a saline solution (0.85% NaCl), followed by turbidity adjustment to match a 0.5 McFarland standard (1.5 × 10^8^ CFU/mL). From these bacterial suspensions, 30 µL were transferred into 125 mL Erlenmeyer flasks containing 30 mL of Ag02 medium (g/L: glucose 15.0; sucrose 10.0; yeast extract 10.0; micronized soybean protein 10.0; KH_2_PO_4_ 1.5; MgSO_4_ 0.5; MnSO_4_ 0.5; and CaCl_2_·2H_2_O 1.5; pH 8.0), and incubated at 30 °C for 18–20 h at 200 rpm in an orbital shaker.

For inoculum production, 1 L Erlenmeyer flasks containing 400 mL of fresh Ag02 medium were inoculated with 4 mL of the pre-inoculum and incubated at 30 °C and 200 rpm for 72 h. After the bioprocess, the final cell concentration was adjusted to 2.0 × 10^9^ CFU/mL.

#### 2.3.2. Field Trials

Field experiments were performed using soybean cultivar Credenz Result I2X (BASF^®^, BASF, Ludwigshafen, Germany) seeds. Seeds were individually treated with the biological products Ag87, Ag94, Ag87 + Ag94, or the commercial product (CNPMS B119 + CNPMS B2084) in plastic bags, using a dose of 100 mL per 50 kg of seeds. The control treatments consisted of non-inoculated seeds combined with three different phosphorus (P) fertilizer rates applied to the soil (25 kg P_2_O_5_, 42 kg P_2_O_5_, and 84 kg P_2_O_5_ for ha). For the treatments inoculated with bacterial strains, the lowest P dose (25 kg P_2_O_5_) was used. A treatment without phosphate fertilization was not included, as this practice is uncommon among Brazilian farmers due to the naturally low phosphorus availability in most Brazilian agricultural soils.

The experiments were carried out during the 2020/2021 and 2021/2022 growing seasons in two municipalities in Paraná, Brazil: Londrina (23°18′36″ S, 51°9′46″ W) and Guarapuava (25°23′0″ S, 51°27′0″ W). According to Köppen climate classification, both places present humid subtropical climate, differing by the level of heat, respectively Cfa and Cfb.

The experimental design was a randomized complete block with four replicates. Each plot consisted of eight rows, 6 m long, spaced 0.45 m apart. Before trial establishment, the areas were fertilized with 25 kg P_2_O_5_/ha and 60 kg K_2_O/ha. The applied P_2_O_5_ rates corresponded to approximately 30% of the standard P fertilization recommended for soybean cultivation for each place. Fertilization was carried out at sowing, and throughout the experiments, crop management practices were performed, including the application of fungicides, insecticides, and herbicides during crop development.

Grain yield (kg/ha) was determined after the manual harvest of plants from the six central rows of each plot. In all field trials, seeds were inoculated with *Bradyrhizobium japonicum* strains SEMIA 5079 and 5080, using a dose of 100 mL per 50 kg of seed. Phosphorus use efficiency (PUE) components were calculated as the ratio of grain yield (g) to the amount of P applied (g).

### 2.4. Data Analysis

Agronomic data were subjected to analysis of variance (ANOVA) after verifying that model assumptions were met. Treatment means were compared using Tukey’s test (*p* < 0.05). All statistical analyses and graphical visualizations were performed using the R software version 4.4.1 with the AgroR package [32].

## 3. Results

### 3.1. Phytase Production

**Figure 1:**
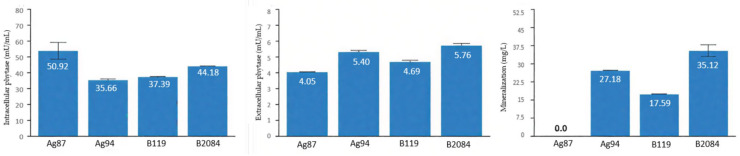
Quantification intra- and extracellular phytase activity and phytate mineralization by bacterial strains cultured in National Botanical Research Institute’s Phosphate (NBRIP) growth medium.

All bacterial isolates evaluated demonstrated the ability to produce phytase, with the highest concentrations observed in the intracellular fraction (Figure 1). Due to the exploratory nature of this assay, statistical comparisons were not conducted. For intracellular phytase, the highest activities were recorded for strains Ag87 and B2084 (50.92 and 44.18 mU/mL, respectively). In contrast, the highest values for extracellular phytase were observed in strains B2084 and Ag94 (5.75 and 5.40 mU/mL, respectively). No phytate mineralization was detected for strain Ag87, while strains Ag94, B119, and B2084 exhibited mineralization values of 27.18, 17.59 and 35.12 mg/L, respectively.

### 3.2. Acid and Alkaline Phosphatase Production

**Figure 2:**
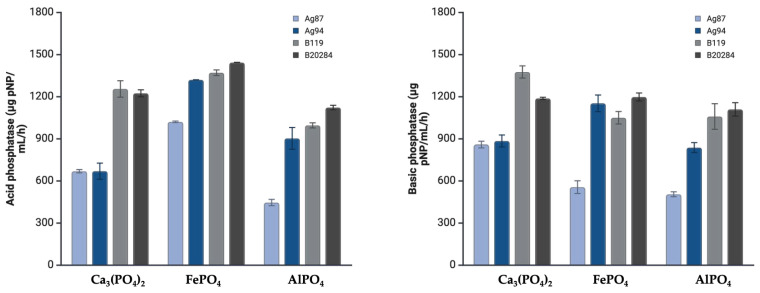
Quantification of acid and alkaline phosphatase activity in bacterial strains cultured in National Botanical Research Institute’s Phosphate (NBRIP) growth medium supplemented with three phosphorus sources (Ca_3_(PO_4_)_2_, FePO_4_, and AlPO_4_).

All bacterial strains exhibited the ability to produce both acid and alkaline phosphatases when cultured with the three phosphate sources (Ca_3_(PO_4_)_2_, FePO_4_, and AlPO_4_) (Figure 2). For acid phosphatase, the highest mean activity was observed with FePO_4_ as the P source, reaching 1290.67 µg pNPP/mL/h. Strains B119 and B2084 showed the highest enzyme activity across all phosphate sources and exhibited activity for all the three P sources. For alkaline phosphatase, the highest mean activities were recorded with Ca_3_(PO_4_)_2_ and FePO_4_ as phosphorus sources (1062.5 and 1016.24 µg pNPP/mL/h, respectively).

Meanwhile, strains Ag87 and Ag94 exhibited considerable mean activities of phosphatases. The highest activity of acid phosphatase (1240.00 µg pNPP/mL/h) with Ca_3_(PO_4_)_2_ and highest activity of basic phosphatases with Fe(PO_4_), both produced by Ag94 (CCT 8108).

Considering strains Ag87 and Ag94, the highest mean activity for acid phosphatases were obtained with Fe(PO_4_), whilst highest mean activity of basic phosphatase was obtained with Ca_3_(PO_4_)_2_. Nonetheless, basal phosphatase activity was recorded sustaining a minimum of 400 µg pNPP mL^−1^ h^−1^ for Ag87, but capable of reaching up to 900 µg pNPP mL^−1^ h^−1^ when considered with Al(PO_4_) in both cases.

### 3.3. Organic Acid Production

**Figure 3:**
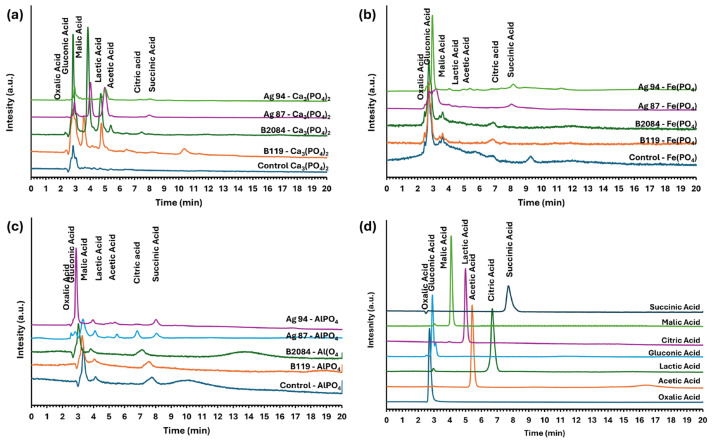
Chromatogram of organic acids produced by bacterial strains (Ag87 (CCT 8090), Ag94 (CCT 8108), B119, and B2084) cultured in National Botanical Research Institute’s Phosphate (NBRIP) growth medium supplemented with three phosphorus sources: (**a**) Ca_3_(PO_4_)_2_; (**b**) FePO_4_; (**c**) AlPO_4_; and (**d**) organic acid standards.

Organic acid production varied among the bacterial strains and according to the P source used (Table 1; Figure 3). In the presence of Ca_3_(PO_4_)_2_, as the sole phosphorus source, strain Ag87 showed the highest total organic acid production (387.53 mmol/L), predominantly lactic acid (342.43 mmol/L), followed by malic and acetic acids (18.02 and 18.00 mmol/L, respectively). Strain Ag94 exhibited a comparatively lower total production (139.98 mmol/L), also dominated by lactic acid (123.85 mmol/L). Strain B119 produced the highest level of gluconic acid (73.53 mmol/L), along with lactic and malic acids (21.31 and 20.65 mmol/L, respectively), totaling 121.56 mmol/L. Strain B2084 showed moderate malic, lactic, and gluconic acid production (53.52, 35.82, and 28.17 mmol/L, respectively), totaling 131.37 mmol/L.

When FePO_4_ and AlPO_4_ were used as P sources, total acid production decreased for all strains. Under FePO_4_ source, strain Ag94 showed the highest total acid production (20.80 mmol L^−1^), mainly comprising gluconic, acetic, and lactic acids (7.59, 5.60, and 5.17 mmol/L, respectively). Strain Ag87 produced notable amounts of gluconic and succinic acids (6.25 and 3.80 mmol/L, respectively), while strain B119 mainly produced gluconic acid (15.60 mmol/L). No detectable production of the target organic acids was observed for strain B2084 under the tested conditions. In the presence of AlPO_4_, only strains Ag87 and Ag94 produced detectable levels of organic acids. Strain Ag87 primarily secreted acetic acid (50.96 mmol/L), totaling 57.74 mmol/L, whereas Ag94 reached 19.58 mmol/L, with significant contributions from gluconic and acetic acids.

### 3.4. Field Trials

Analysis of variance revealed significant effects of treatment and environment on grain yield and PUE (Table 2). A significant treatment × environment interaction was observed only for PUE. Among the environments, Guarapuava (2021/2022) showed the highest mean yield (4079.41 kg/ha), followed by Londrina (2021/2022) (3375.62 kg/ha), Guarapuava (2020/2021) (3074.65 kg/ha), and Londrina (2020/2021) (3058.95 kg/ha). Among treatments, the control with 84 kg P_2_O_5_/ha showed the highest yield (3651.21 kg/ha), but was not statistically different from the treatments Ag87 + Ag94 (3572.85 kg/ha), Ag94 (3540.44 kg/ha), the control with 42 kg P_2_O_5_/ha (3417.91 kg/ha), or from the commercial standard product (B119 + B2084) (3303.20 kg ha^−1^) (Table 3).

PUE was defined as the ratio between grain dry biomass and the amount of P applied. All treatments inoculated with bacterial strains received 25 kg P_2_O_5_/ha, corresponding to 30% of the recommended P dosage for soybeans. To ensure a meaningful comparison, the inoculated treatments were evaluated against the non-inoculated control, receiving the same P doses (25 kg P_2_O_5_/ha) since the controls with higher P inputs (42 and 84 kg P_2_O_5_/ha), and naturally resulting in lower PUE ratios due to the greater amount of P applied. In the Londrina (2020/2021) environment, the highest PUE values were observed for the (B119 + B2084) treatment, followed by Ag94 and Ag87 (Table 2). In Guarapuava (2020/2021), no statistical differences were detected among the inoculated treatments, all of which outperformed the non-inoculated control, with 25 kg P_2_O_5_/ha. In Londrina (2021/2022), no significant differences were observed between the inoculated treatments and the control, whereas in Guarapuava (2021/2022), the highest PUE values were recorded for treatments Ag87 and Ag87 + Ag94.

## 4. Discussion

PSMs have been extensively studied [19] and are increasingly incorporated into agricultural systems as a strategy to enhance the efficiency of phosphate fertilizers [10,11,20]. Although soils contain significant reserves of total P, it is estimated that up to 80% of this nutrient is immobilized in insoluble inorganic forms or complexed with organic matter, rendering it unavailable for plant uptake [20,26]. To overcome this limitation, PSMs mobilize P through direct and indirect mechanisms. Among the direct mechanisms, the production of organic acids is particularly important, as these compounds chelate cations such as Fe^3+^, Al^3+^, and Ca^2+^, thereby releasing soluble orthophosphate into the rhizosphere [19]. In addition, these microorganisms produce enzymes such as phosphatases and phytases, which hydrolyze organic P compounds into inorganic, plant-available forms [20,26]. Phytase activity in the bacterial strains indicates their potential to mineralize organic P in the soil, particularly in phytate (phytic acid). Phytate is the primary form of organic P in soils; however, its stability and strong binding to metal cations limit its availability to plants [24,33]. For this P to become plant-accessible, phytate must be hydrolyzed, primarily by microbial phytases, resulting in the release of inorganic orthophosphate and inositol phosphate derivatives, which can subsequently be further degraded by acid and alkaline phosphatases.

The intra- and extracellular phytase activities and the phytate mineralization values observed in the present study are consistent with those reported by Oliveira-Paiva [27], with intracellular phytase predominant. The prevalence of intracellular phytase may be associated with the internal metabolic demands of the microorganisms [24,34]. However, this intracellular form is limited in soil phytate mineralization, as the substrate is in the extracellular environment. In this regard, strains B2084 and Ag94 (CCT 8108) stood out for their extracellular phytase activity and ability to mineralize phytate. Idriss et al. [35] demonstrated that the extracellular phytase produced by *B. amyloliquefaciens FZB45* exhibited catalytic activity toward phytate hydrolysis, directly contributing to the release of inorganic P. When the enzyme-containing filtrate was applied to maize seedlings, it enhanced root growth and increased chlorophyll content, highlighting the role of phytase in improving P nutrition and promoting plant development.

Phosphatases also play a crucial role in mineralizing organic P compounds, acting as mediators in releasing orthophosphate from ester-bound P molecules [21]. For all four bacterial strains evaluated, phosphatase activity varied depending on the phosphate source. These variations are likely related to differences in solubility, binding affinity, and feedback regulation mechanisms associated with P availability. Strains B119 and B2084 exhibited the highest acid and alkaline phosphatase activities across all three P sources that supplemented NBRIP medium, highlighting its own P metabolic flexibility and adaptive capacity.

The variability in organic acid production among the evaluated bacterial strains and its dependence on the P source indicates a metabolically regulated response highly sensitive to environmental conditions. In the presence of tricalcium phosphate, strains Ag87 and Ag94 accumulated higher amounts of lactic acid, with lower concentrations of acetic, malic, and gluconic acids. This metabolic pattern suggests the predominance of homolactic fermentation, a typical pathway in Gram-positive bacteria, in which glucose is converted primarily into lactic acid. These findings are consistent with those reported by Mazhar et al. [36], who demonstrated that *B. coagulans* predominantly produce lactic acid, a trait associated with efficient acidification of the growth medium, a critical condition for calcium phosphate solubilization.

Strains B119 and B2084 exhibited more diverse metabolic profiles, with notable gluconic, malic, and acetic acid production. Gluconic acid is associated with phosphate solubilization via cation complexation. It is widely reported as one of the most efficient P-release mechanisms, particularly in media containing calcium, iron, and aluminum phosphates [36,37]. Although strains Ag87 and B119 belong to the same species (*Priestia megaterium*), they exhibited distinct organic acid production profiles, highlighting the influence of strain-specific genetic and regulatory factors on the expression of metabolic pathways related to phosphate solubilization. This type of intraspecific variation was also reported by Vyas and Gulati [38], who demonstrated significant differences in acid production among *Pseudomonas* strains isolated from different environments.

For the organic acids analyzed, metabolic responses to iron and aluminum phosphates were more limited, indicating an inhibitory effect of metal ions on organic acid metabolism. This interference may be related to impaired enzymatic activity, as Fe^3+^ and Al^3+^ can compete with essential cofactors such as Mg^2+^ and Mn^2+^, disrupting the biosynthetic pathways responsible for organic acid production. Moreover, Fe^3+^ and Al^3+^ may induce oxidative stress, redirecting bacterial metabolism toward antioxidant defense pathways and consequently limiting the biosynthesis of secondary metabolites, including organic acids [38,39,40].

In the presence of FePO_4_ and AlPO_4_, strains Ag87 and Ag94 produced a more diverse array of organic compounds, which may represent an adaptive strategy to the chemical complexity of these phosphate forms. Unlike Ca_3_(PO_4_)_2_, which can be solubilized through direct acidification of the medium, FePO_4_ and AlPO_4_ require additional mechanisms, such as selective chelation of metal cations by organic acids containing a greater number of carboxyl or hydroxyl groups, such as gluconic and malic acids [37,38]. The combined production of different organic acids enhances the ability of bacteria to form soluble complexes with Fe^3+^ and Al^3+^, thereby releasing previously precipitated P. This metabolic versatility also enables broader activity across diverse soil conditions, supporting the application of these strains as bioinoculants in soils with high P fixation by iron and aluminum, a common condition in highly weathered tropical regions.

Strains Ag87 and Ag94 were previously validated in maize, demonstrating their potential as plant growth-promoting and phosphate-solubilizing bacteria. In soybeans, we also observed promising results for the combined use of these strains, with a 15.34% increase in yield compared to the control receiving 30% of the recommended P dose and similar productivity to treatments receiving 50% and 100% P fertilization. Notably, strain Ag94 also showed strong performance when applied individually, significantly increasing yield. Ag94 is a *Lysinibacillus* sp., likely a novel species within the genus [10]. Strains of *Lysinibacillus* sp. have been studied for similar purposes, with promising results supporting their use as bioinoculants [41,42,43,44]. Pan et al. [43] found that *L. sphaericus* M1 was effective in phosphate solubilization, particularly through the secretion of malic and succinic acids and the enhancement of soil phosphatase activity, which led to increased P uptake and plant growth in rice under P-deficient conditions. Vitorino et al. [44] demonstrated that combining *L. fusiformis* and *B. velezensis* promoted root system development, increased nodule number and dry mass, and improved soybean yield. These effects were attributed to the release of organic acids, phytase production, and auxin synthesis.

The higher PUE observed in treatments inoculated with bacterial strains may be attributed to their ability to solubilize and mineralize soil P and their direct effect on root growth stimulation. Plants inoculated with plant growth-promoting bacteria (PGPB) often develop multiple adaptive strategies to optimize nutrient uptake, including modulation of gene expression related to iron transport, increased root biomass, and enhanced root system architecture, factors that improve soil exploration and intensified rhizosphere interactions, which facilitate the mobilization and availability of essential nutrients [42,44].

## 5. Conclusions

The bacterial strains of both commercial products Nodubiophos^®^ and Biomaphos^®^ demonstrated functional potential for P solubilization and mineralization through phytase, phosphatases, and carboxylic organic acids. Strains B2084 and Ag94 stood out for their phytate mineralization capacity, while B119 and B2084 showed the highest acid and alkaline phosphatase activities. The phosphate source modulated organic acid production by the bacterial strains, with strain Ag87 showing greater activity in the presence of Ca_3_(PO_4_)_2_ and AlPO_4_, and Ag94 in the presence of FePO_4_.

In field trials, the combination of strains Ag87 and Ag94 enhanced soybean yield, achieving performance comparable to conventional fertilization with 50 and 100% of the recommended P dose, even when only 30% of the recommended P was applied. Also, strain Ag94 alone exhibited promising results, highlighting its potential as a bioinoculant for improving PUE in soybean cultivation, even in places where high chemisorption of phosphorous occurs.

## Figures and Tables

**Table 1:** Quantification of organic acids (mmol L^−1^) produced by bacterial strains cultured in National Botanical Research Institute’s Phosphate (NBRIP) growth medium supplemented with different phosphorus sources (Ca_3_(PO_4_)_2_, FePO_4_, and AlPO_4_). ND: Not detected.

Organic Acids	Strains			
	Ag87 (CCT 8090)	Ag94 (CCT 8108)	B119	B2084
Ca_3_(PO_4_)_2_				
Gluconic	6.37	6.16	73.53	28.17
Malic	18.02	0.47	20.65	53.52
Lactic	342.43	123.85	21.31	35.82
Acetic	18.00	8.11	6.07	11.01
Citric	ND	ND	ND	ND
Succinic	2.71	1.39	ND	2.86
Total	387.53	139.98	121.56	131.37
FePO_4_				
Gluconic	6.25	7.59	15.6	ND
Malic	1.25	0.48	0.92	ND
Lactic	1.07	5.17	ND	ND
Acetic	0.82	5.6	ND	ND
Citric	ND	0.17	ND	ND
Succinic	3.8	1.79	ND	ND
Total	13.19	20.80	16.52	ND
AlPO_4_				
Gluconic	0.91	6.43	ND	ND
Malic	1.55	0.71	ND	ND
Lactic	1.7	3.43	ND	ND
Acetic	50.96	8.93	ND	ND
Citric	0.74	0.07	ND	ND
Succinic	1.88	0.01	ND	ND
Total	57.74	19.58	ND	ND

**Table 2:** Analysis of variance for yield and phosphorus use efficiency in soybean experiments using seeds inoculated with different phosphate-solubilizing bacteria.

Source of Variation	DF	Mean Square ^1/^	
		Yield	Phosphorus Use Efficiency (PUE)
Repetitions/E	12	141,508.7	1790.45
Treatments (T)	6	680,907.6 **	57,238.6 *
Environment (E)	3	6,390,671.4 **	92,850.06 **
T × E	18	327,153.4 ^ns^	5829.55 *
Error	72	209,639.5	1126.27
CV(%)		13.47	21.66
Means			
Londrina (20/21)		3058.95	127.89
Guarapuava (20/21)		3074.05	153.56
Londrina (21/22)		3375.62	102.71
Guarapuava (21/22)		4079.41	235.48

^1/^ ns: not significant; * and **: significant at 5% and 1% probability levels by the F-test, respectively. DF: Degrees of Freedom.

**Table 3:** Tukey’s test (*p* < 0.05) for grain yield and phosphorus use efficiency in soybean experiments conducted during the 2020/2021 growing season using seeds inoculated with different phosphate-solubilizing bacteria.

Treatments	Yield ^1/^		Phosphorus Use Efficiency (PUE)			
	Mean	Δ%	Londrina (20/21)	Guarapuava (20/21)	Londrina (21/22)	Guarapuava (21/22)
Ag87 (CCT 8090)	3196.15 bc	3.18	156.77 abc	183.71 ab	97.98 bc	357.89 a
Ag94 (CCT 8108)	3540.44 ab	14.30	176.37 ab	182.21 ab	169.01 a	292.21 b
Ag87 + Ag94 (CCT 8090 + CCT 8108)	3572.65 a	15.34	134.11 bc	200.87 a	117.57 b	331.80 ab
Commercial standard (B119 + B2084)	3303.20 abc	6.64	187.39 a	213.11 a	97.98 bc	224.22 c
25 Kg P_2_O_5_	3097.47 c		129.83 c	163.50 b	128.60 ab	220.27 c
42 Kg P_2_O_5_	3417.91 abc		76.05 d	80.47 c	67.60 cd	130.99 d
84 Kg P_2_O_5_	3651.21 a		34.72 d	40.60 c	40.23 d	90.98 d

^1/^ Means followed by the same letter in the same column do not differ significantly according to Tukey’s test (*p* < 0.05).

## Data Availability

The original contributions presented in this study are included in the article. Further inquiries can be directed to the corresponding authors.
